# Evaluating use of web-based interventions: an example of a Dutch sexual health intervention

**DOI:** 10.1093/heapro/daab190

**Published:** 2021-11-22

**Authors:** Gido Metz, Hanneke Roosjen, Wessel Zweers, Rik Crutzen

**Affiliations:** 1 Department of Health Promotion, Maastricht University/CAPHRI, The Netherlands; 2 Soa Aids Nederland, Amsterdam, The Netherlands

**Keywords:** web-based intervention, eHealth, acyclic behavior change diagrams, web analytics, evaluation

## Abstract

With the current increase in web-based interventions, the question of how to measure, and consequently improve engagement in such interventions is gaining more importance. Modern day web analytics tools make it easy to monitor use of web-based interventions. However, in this article, we propose that it would be more meaningful to first examine how the developers envisioned the use of the intervention to establish behavior change (i.e. intended use), before looking into how the intervention is ultimately used with web analytics (i.e. actual use). Such an approach responds to the regularly expressed concern that behavioral interventions are often poorly described, leading to less meaningful evaluations as it is not clear what exactly is being evaluated. Using a page on chlamydia prevention (104 557 pageviews in 2020) from a Dutch sexual health intervention (Sense), we demonstrate the value of acyclic behavior change diagrams (ABCDs) as a method to visualize intended use of an intervention. ABCDs show at a glance how behavior change principles are applied in an intervention and target determinants of behavior. Based on this ABCD, we investigate actual use of the intervention, using web analytics tool Matomo. Despite being intended to stimulate STI-testing, only 14% of the 35 347 transfers from this page led to the STI-testing page and a high bounce rate (79%) and relatively high exit rate were reported (69%). Recommendations to further interpret the data are given. This real-life example demonstrates the potential of combining ABCDs and Matomo as methods to gain insight into use of web-based interventions.

## INTRODUCTION

Over the years, a rising number of web-based interventions have been developed that target various behaviors, such as safer sex behaviors (Bailey *et al.*, 2012). Generally, web-based interventions are effective, but their use is limited (Kohl *et al.*, 2013), which in turn limits the public health impact of such interventions (Glasgow *et al.*, 2019). The question of how to measure, and consequently improve engagement in web-based interventions, is therefore gaining more importance (Short *et al.*, 2018). A review on web-based interventions shows that automatic tracking of behavior is one of the most commonly used measures of engagement (Perski *et al.*, 2017b). Web analytics tools like Google Analytics and Matomo make it easy indeed to monitor use of web-based interventions and they provide detailed overviews of use data such as log-ins, average time spent on (parts of) the intervention and paths taken during a visit (Crutzen *et al.*, 2013).

However, investigating use data should not be an isolated step in evaluating web-based interventions. Instead of looking directly at actual use, it can be more meaningful to first examine how the developers envisioned the use of the intervention to establish behavior change (i.e. intended use), apart from how the intervention is ultimately used (i.e. actual use). Such an approach responds to the regularly expressed concern that behavioral interventions are often poorly described, leading to less meaningful evaluations as it is not clear what exactly is being evaluated (Kok and Mesters, 2011; Albrecht *et al.*, 2013; Abraham *et al.*, 2014). So, a more comprehensive insight into the impact of the intervention will be reached when there is a clear overview of the intervention (e.g. what the intervention entails and how it is hypothesized to lead to behavior change) and when use data are analyzed in connection with this overview.

An overview of an intervention is also referred to as a logic model of change (Bartholomew Eldredge *et al.*, 2016). As interventions often combine multiple behavior change principles and address multiple determinants and sub-behaviors, an overwhelming logic model can quickly arise. Acyclic Behavior Change Diagrams (ABCDs) show at a glance which causal-structural relationships underlie a behavioral intervention (Peters and Crutzen, 2021). In this article, we show the potential of ABCDs and how they can be of help in evaluating interventions with web analytics tools.

As an example, we will be using data from the Dutch sexual health website Sense (www.sense.info; last accessed: 4 October 2021). Sense is a crucial element of the national eHealth strategy and the website serves as the entrance point for youngsters in the Netherlands to prevention (and care if needed) regarding the whole spectrum of sexual and reproductive health. The website has been online since 2009, and the number of visits grew from 643 540 in the first year to 6 269 298 in 2020. Sense has also been included in the Dutch recognition system for health promotion interventions (Brug *et al.*, 2010).

## INTENDED USE: ABCDs

In the previous section, ABCDs were described as a detailed visualization of a logic model of change, with a reference to the Intervention Mapping approach (Bartholomew Eldredge *et al.*, 2016). However, ABCDs can be used regardless of which planning framework is used and is independent of Intervention Mapping as such. They visualize how behavior change principles from multiple theories are applied in an intervention and target determinants of behavior (Crutzen and Peters, 2019; Peters and Crutzen, 2021).

**Fig. 1:**
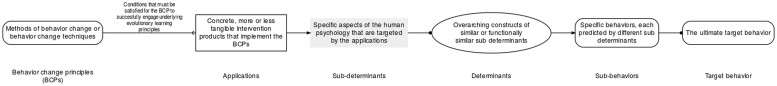
A simple overview of a causal-structural chain of an ABCD. Note. Each box represents one of the seven columns in the ABCD matrix. The text in the boxes gives an explanation of each column (based on Crutzen and Peters, 2019). The lines with the fork-shaped ends in between behavior change principles and application stand for ‘implemented in’. Applications are manifest constructs and have, in the tradition of drawing pathways in structural equation modeling (SEM; Stein *et al.*, 2012), been placed in rectangles. In turn, the determinants have been placed in ovals as they are latent constructs (again in the tradition of SEM). Lines with dotted ends between sub-determinants and determinants and between sub-behaviors and the target behavior have been used to indicate a causal relationship. The shape of the boxes for sub-determinants have been chosen to distinguish them from the rest, as they are less likely to be conceptualized as a latent construct on their own.

Creating an ABCD starts by making an ABCD matrix. This is a table with seven columns, where each row represents one causal-structural chain. The assumed causal associations run in one direction and there are no loops, hence the acyclic nature of the ABCD. In other words, the intervention targets (sub-)determinants of behavior to ultimately result in behavior change. Therefore, the seventh and last column of the chain is the target behavior. In the six columns preceding the target behavior, it is described (i) which behavior change principles are applied—(ii) taking into account the parameters for use—in (iii) a specific application, which (iv) sub-determinants and (v) determinants are addressed and (vi) which sub-behaviors should be executed to reach the target behavior. In Figure 1, we give an overview of a causal-structural chain, including short explanations of each column.

When the ABCD matrix of the intervention has been completed, an overview has been created of all elements (e.g. included applications) that the intervention combines. This can result in a matrix that has expanded considerably. ABCDs visually represent the seven columns of the ABCD matrix, but with cells with the same content merged to be represented by the same element (Crutzen and Peters, 2019; Peters and Crutzen, 2021). By visually merging duplicated elements of the structural-causal chains in the ABCD matrix, it is easier to get an overview of the logic model represented in the ABCD matrix (and underlying the intervention). Although ABCDs can get rather big, we believe they are the simplest possible overview of an intervention that at the same time shows all structural-causal chains underlying the intervention. Where the matrix can be used to focus on specific details of the intervention, the ABCD is useful in visualizing all structural-causal relationships and uncovering missing links. ABCDs are also easy to adjust according to one’s own wishes; in case a smaller ABCD is desired, separate ABCDs for specific parts of the intervention can be made. As such, we expect that ABCDs are created by and very useful when communicating about how an intervention (is intended to) work(s) with colleagues, members of an intervention planning group, or other parties, such as the executive intervention producers. This also facilitates them to grasp the complexity underlying what might come across as a simple intervention (e.g. a website).

### ABCDs applied: chlamydia prevention on Sense

**Fig. 2:**
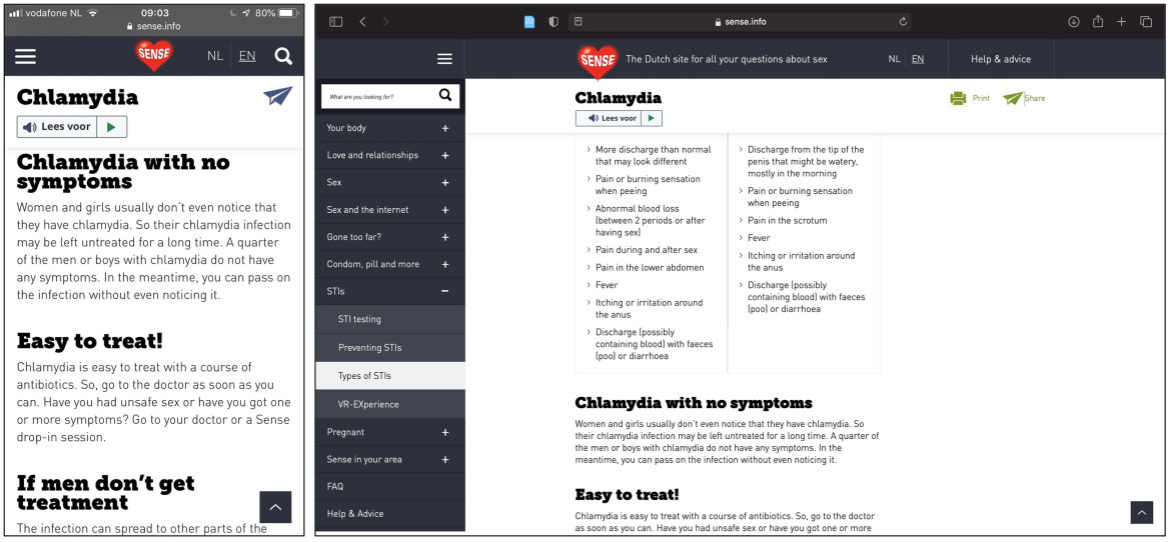
Screenshots of a part of Sense’s chlamydia page (left: smartphone view, right: desktop view). Note. We specifically focused on the Dutch version of Sense’s chlamydia page in our analysis. However, we decided to provide screenshots of the English version of the page for clarity.

We will now give an example of an ABCD for a frequently visited page of Sense; the page about chlamydia (Figure 2). This ABCD—and the analysis of use data that we will describe in the next section—obviously focuses specifically on Sense. However, the underlying ideas and techniques are generic in nature and can be applied in different contexts as well. So, in this example, we created an ABCD of an existing intervention. We therefore closely checked with the intervention developer whether the ABCD reflects the true intentions of the developers to ensure validity of the ABCD.

The overarching target behavior of the chlamydia page is to ‘have sex without STIs’. Several sub-behaviors can be distinguished on this page, such as ‘using condoms’, ‘getting tested’, ‘seeking treatment’ and ‘warning your partner(s) if you have an STI’. Multiple behavior change principles, based on different underlying theories, are being used in different practical applications to change sub-determinants and determinants to help promote executing the sub-behaviors and, ultimately, the target behavior. We adhere to the taxonomy of behavior change principles by Kok *et al.* (2016) to define the relevant behavior change principles and underlying parameters for use. For example, we concluded that the principle ‘consciousness raising’, based on the Health Belief Model (Skinner *et al.*, 2015), had been used by means of providing textual information about the causes, consequences and possible means of prevention of chlamydia. Several determinants have been addressed, which we will set out in the next paragraph.

As the screenshot of the page in Figure 2 shows, there is a bulleted overview of the different symptoms of chlamydia (broken down by gender), addressing the determinant knowledge. The two paragraphs below this overview address several determinants:

*Women and girls usually don’t even notice that they have chlamydia. So, their chlamydia infection may be left untreated for a long time. A quarter of the men or boys with chlamydia do not have any symptoms. In the meantime, you can pass on the infection without even noticing it.*

This paragraph addresses the determinants knowledge and risk perception, as it points out the possibility of not noticing a chlamydia infection and therefore passing it on to sexual partners, also implying that the reader might have been infected without even noticing. Subsequently, the second paragraph informs about the relative ease with which chlamydia can be cured and that you should visit your general practitioner or Sense consultation hour:

*Chlamydia is easy to treat with a course of antibiotics. So, go to the doctor as soon as you can. Have you had unsafe sex, or have you got one or more symptoms? Go to your doctor or a Sense drop-in session.*

This paragraph addresses both beliefs underlying attitudes (‘I should visit my general practitioner/Sense consultation hour if I have symptoms’) as well as self-efficacy (‘it is easy for you to get treated’). The combination of raising awareness about the symptoms and consequences of a chlamydia infection and expressing confidence that chlamydia is easy to cure means that one of the parameters of consciousness raising has been met, namely that raising awareness must be quickly followed by increase in problem-solving ability and self-efficacy. However, we believe it might have been a valuable addition to also target the self-efficacy belief that getting tested is very easy and convenient. Visiting a general practitioner or Sense consultation hour for an STI-test if needed is one of the most important goals of this page and it is likely that young people experience psychological barriers to visit a doctor for this (Cuffe *et al.*, 2016). We expect that not addressing this barrier might influence the potential impact of the intervention on the sub-behavior of getting tested.

**Fig. 3:**
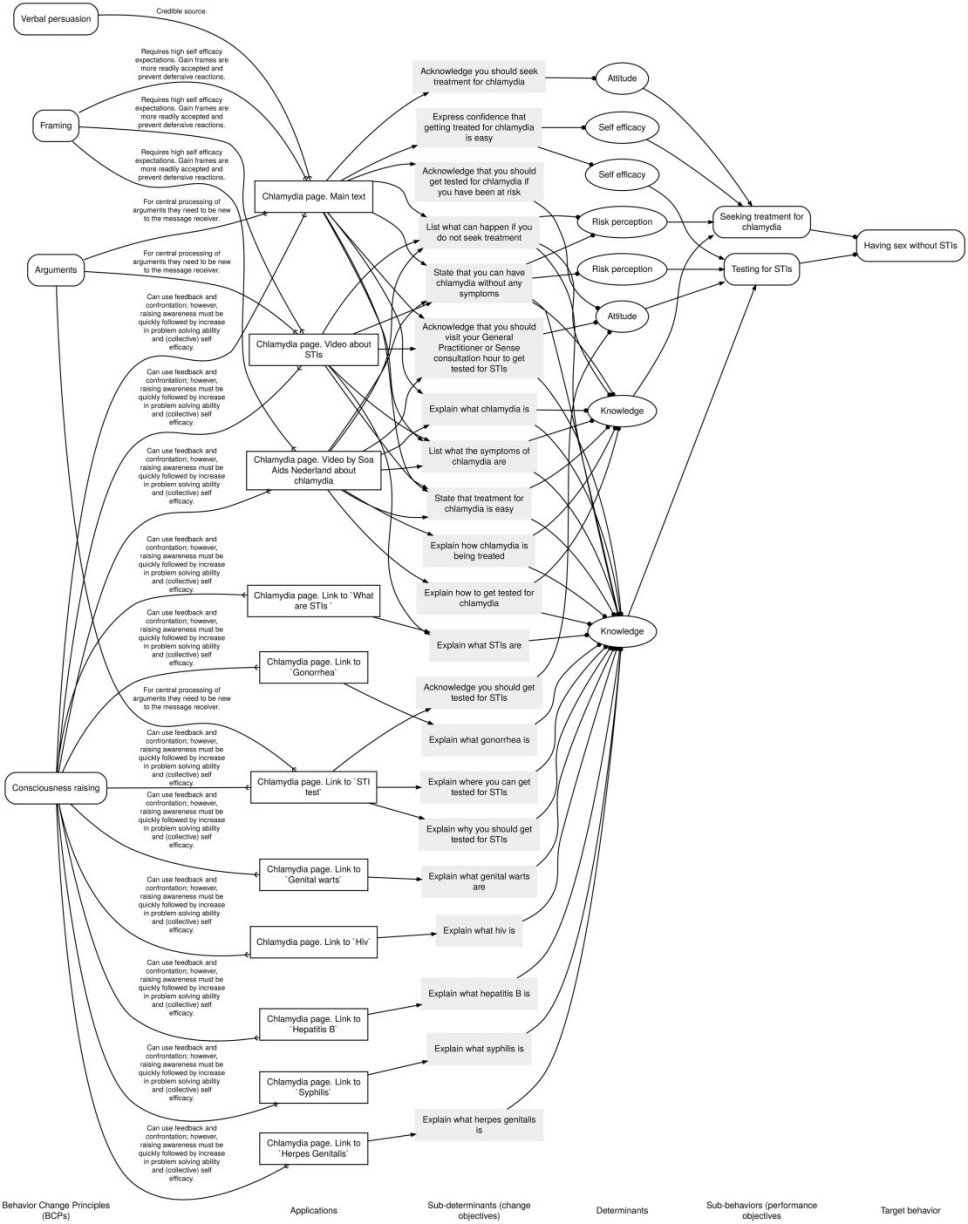
Excerpt from the ABCD for Sense’s page on chlamydia.

Integration of all the used behavior change principles and applications eventually lead to the ABCD matrix in Supplementary File SA and the ABCD in Supplementary File SB (for the sake of readability, we have included an excerpt of this ABCD in Figure 3). This ABCD also shows us that the page on chlamydia is quite an extensive page; besides the textual information provided above, there are several video clips and click-through possibilities, most of them with the goal of motivating people to get tested for STIs. Based on this ABCD, we have an overview of how the intervention was intended to be used. Besides that, the previous paragraph shows that creating the ABCD has already led us to come up with a first expectation on (where there is room for improvement regarding the) potential impact of the intervention. In the next paragraph, we will show how web analytics provide insight into how intended use works out in practice.

## COMBINING INTENDED USE AND ACTUAL USE: WEB ANALYTICS

After obtaining an overview of the intended use of the intervention by means of the ABCDs, they can be combined with insight into actual use, based on web analytics. The combination of actual use of the website and intended use provides a comprehensive picture on which elements of an intervention are used as intended and which paths are taken within each visit. This not only shows the relationship between elements, but also where additional elements might be needed and which elements need improvement to reach the aims of the intervention.

In the case of Sense, we use in-depth metrices that are available in Matomo to obtain data on actual use of the intervention. Matomo is a secure open-source web analytics platform, and adheres to current standards of data protection in line with the European General Data Protection Regulation (Crutzen *et al.*, 2019; Quintel and Wilson, 2020; Matomo, 2021). Matomo provides insight into several metrices [e.g. keywords used to reach certain elements, time spent using these elements, order in which certain elements are used (Ripp and Falke, 2018)], that, when taken together, do not only allow to distinguish between use/non-use, but also give insight into the quality of use (e.g. do visitors of an information page about use of condoms also click on the link to the condom shop and buy condoms?). In other words, a more comprehensive picture of actual use of elements. Although Matomo is primarily intended as an analytics tool for websites (on both desktop and mobile platforms), the underlying method of analyzing data that we describe here is also relevant for mobile app-based interventions and any other digital form of intervention with a possibility to track use data [e.g. Piwik PRO (https://piwik.pro; last accessed: 4 October 2021) could be used as an analytics tool for app-based intervention].

Prior to the analysis, we made two decisions regarding data inclusion. The first decision related to the fact that the website is also visited from abroad (partly due to the English version of the website, which has international students in the Netherlands as its target group). We decided to only include data from visitors from the Netherlands. This has to do with the underlying stepped care model of Sense. Visitors from abroad can of course look up information on Sense, but it is more difficult for them to go through the various steps in the model and, for example, attend the Sense consultation hour within the Dutch public health system. In order to be able to only analyze data from visitors from the Netherlands, we used the option in Matomo to create segments. The second decision was about the date range. Sense has been using Matomo since December 2017, so data were available from that point on. However, Matomo did not currently have the capacity to generate data over 3 years. We have therefore chosen to only include data for the year 2020. We did cross-check whether the patterns we observed in 2020 were similar to those in previous years (i.e. 2018 and 2019)—this was indeed the case.

### Incoming traffic

Matomo shows that the page on chlamydia is viewed very regularly; in 2020, the page was in 11th place in the list of most visited pages of Sense and it was the most visited page of Sense about STIs. Most visitors reach the page after a search on Google (Table 1). There seems to be a focus on symptoms in the searches (*n* = 54 066), as the most used search queries in Google were ‘chlamydia’ (14.6%), ‘chlamydia symptoms’ (12.2%), ‘symptoms chlamydia’ (6.3%), ‘chlamydia symptoms woman’ (3.8%), ‘symptoms chlamydia woman’ (3.4%) and ‘symptoms chlamydia man’ (2.0%). A somewhat smaller part of the visitors reached the page through internal pages (*n* = 37 287); mainly via a general referral page with links to different STIs (‘types of STIs’; 58%), followed by a page with a general explanation of STIs (‘what are STIs?’; 8.1%) and pages about specific STIs (‘gonorrhea’, 3.9%; ‘candida infection’, 3.6%; ‘bacterial vaginosis’, 2.8%). Internal searches led to the page about chlamydia 262 times. Visitors mainly searched for ‘Chlamydia’ (32%) or ‘chlamydia’ (21%), followed by ‘treatment chlamydia’ (1.5%), ‘sti’ (1.5%) or ‘Sti’ (1.5%;the search queries mentioned in this and the next paragraph have been translated from Dutch into English).

**Table 1:** Incoming traffic to and outgoing traffic from Sense’s chlamydia page (2020)

		Count	Percentage
Incoming traffic	Search engines	54.066	54.4
	Internal pages	37.287	37.5
	Direct visits	5.884	5.9
	Other websites	1.874	1.9
	Internal searches	262	0.3
	Social networks	2	0.0
Outgoing traffic	Internal pages	35.347	35.5
	Internal searches	375	0.38
	Outlinks (to other websites)	150	0.15
	End of visit	63.667	64

These data on incoming traffic do not show directly that the intervention is successful in motivating the overarching target behavior or sub-behaviors. However, they do provide first insights into what visitors were looking for when visiting the chlamydia page. A large portion of visitors were looking for information about symptoms of chlamydia. It is likely that they visited the right page; the ABCD shows that listing the symptoms of chlamydia was one of the sub-determinants of this page. Possibly, these visitors might have had certain symptoms and wondered if they could have contracted chlamydia—or another STI, which could account for the portion of visitors that reached the chlamydia page through internal pages like ‘types of STIs’.

### Behavior on the page and outgoing traffic

On average, the page is viewed about 1 min (Table 2). At the same time, however, a high bounce rate (i.e. the percentage of sessions during which visitors left the website after viewing only this page) and a relatively high exit rate are being reported (i.e. the percentage of sessions that finished on this page). Information on page transitions show that of the 35 347 transitions to internal pages, only 14% were to the page about STI-testing (Table 1). The majority (41%) of the transitions were to a general page listing the different types of STIs, followed by pages about gonorrhea (5.6%), genital warts (4.0%) and a page titled ‘what are STIs?’ (4.0%). Information on internal search queries show that ‘Chlamydia’ has been used as a search query in 9.8% of the 375 search queries, followed by ‘gonorrhea’ (6.1%), genital warts (3.2%) and syphilis (2.7%).

**Table 2:** Use data for Sense’s chlamydia page (2020)

Pageviews	Unique pageviews	Bounce rate	Average time spent on page	Exit rate
104 557	91.971	79%	0:01:05	69%

As was also mentioned in the previous paragraph, use data cannot directly show whether the intervention is effective or not; additional research to further interpret use data is always necessary (Perski *et al.*, 2017b; Short *et al.*, 2018). However, the high bounce- and exit rates and the low percentage of transitions to the STI-testing page do raise questions about the impact of this page. Possibly, visitors were just looking for information on (symptoms of) chlamydia—and other STIs—without feeling the need for an STI-test. This assumption could be supported by the finding that the majority of page transitions from this page led to a general page listing the different types of STIs. Another possibility could be that visitors did in fact feel the need for an STI-test after being exposed to the intervention, but went to their general practitioner or made an appointment at the Sense consultation hour in another way than clicking on the link to the STI-testing page. In that case, the intervention would have had impact on the sub-behavior of getting tested and ultimately on the target behavior of having sex without STIs. A last option could be that visitors did not feel capable of getting tested at a Sense consultation hour. This would be in accordance with our previously mentioned observation that not all underlying beliefs of the determinant self-efficacy were addressed with regard to testing for STIs (see section ‘ABCDs applied: chlamydia prevention on Sense’). Possibly, expressing confidence that it is easy to get tested, might have led to more impact with regard to this sub-behavior.

## DISCUSSION

In this article, we demonstrated the potential of ABCDs in describing web-based interventions and how this could lead to more valuable evaluations when combined with web analytics tools such as Matomo. An evaluation of Dutch sexual health intervention Sense (in particular the page on chlamydia) based on intended use and actual use shows us first indications of engagement and the possible impact of the intervention. Several assumptions have been made about how visitors use the page and whether the page leads to behavior change. In the next paragraphs, we will summarize and integrate our findings and make suggestions to further the interpretation of use data and test such assumptions.

In the ABCD for the chlamydia page on Sense, we observed that several determinants were being addressed, such as knowledge, beliefs underlying attitudes and self-efficacy beliefs (We would like to note that we performed this analysis [i.e. created the ABCD] after the intervention had already been developed. Normally, we would recommend performing this analysis before development of the intervention, as it bundles together all matters that need to be considered during development). As regards the latter determinant, self-efficacy beliefs were only addressed when expressing confidence that chlamydia is easy to cure. We reasoned that it would have been a valuable addition to also target self-efficacy beliefs concerning the sub-behavior of getting an STI-test. We assumed that (not) addressing self-efficacy beliefs concerning getting tested might influence the potential impact of the intervention on this sub-behavior, as it is likely that young people experience barriers to see a doctor for an STI-test. These could be, for example, the expectation of financial burden or the fear of embarrassment or being stigmatized (Cuffe *et al.*, 2016).

In the use data analysis, we indeed saw that only a small percentage of the visitors clicked on the link to the page on STI-testing. This could be interpreted as a first indication of evidence in line with the assumption regarding the limited attention to self-efficacy beliefs. However, caution is needed since many other reasons are plausible and more research is needed (as described in more detail in the next paragraph). We also observed that a large percentage of visitors arrived on the page after a Google search for chlamydia symptoms and that another considerable percentage arrived on the page via a general referral page with links to different STIs. This led to multiple assumptions that can be tested in future research. First, visitors might have just been looking for information on STIs out of interest, without having the need for an STI-test. This would be contrary to the expectation also expressed in the ABCD that the page was mainly intended to stimulate STI-testing. Second, it was assumed that visitors might in fact have had the need for an STI-test after being exposed to the intervention but made an appointment at their general practitioner’s or at the Sense consultation hour in another way than clicking on the link to the STI-testing page. In that case, the intervention would have had impact on the sub-behavior of getting tested and ultimately on the target behavior of having sex without STIs. These assumptions can be true for some visitors, but this is not necessarily indicative of a lack of impact. In fact, for some visitors being exposed to information might be sufficient for the time being and be helpful at a later time. The last assumption refers back to visitors feeling the need for an STI-test, but not feeling capable of getting tested and therefore did not click on the link to the page on STI-testing. This would limit the impact of this specific page. Therefore, adding a self-efficacy addressing message like ‘A lot of people like you test frequently for STIs at the Sense consultation hour. Making an appointment is easy, just click on this link’, might have possibly led to more visitors clicking on the link to the page on STI-testing and to more impact of this web-based intervention. A/B tests available in Matomo could be used to test whether such an addition has impact on the percentage of visitors transferring to the STI-test page (Poulos *et al.*, 2020).

In the previous paragraph, we summarized the assumptions that came up during our analysis of intended use with ABCDs and the use data analysis with Matomo. The analysis of use data first showed us that the intervention is being used extensively and second, how it is being used. Although these findings are valuable and use data are regularly used as the sole source of information to measure engagement, this method should not be seen as a valid measurement tool for engagement on its own (Perski *et al.*, 2017b; Short *et al.*, 2018). Analyzing intended use with ABCDs could be a valuable first, additional step. However, after the analysis of actual use, additional qualitative methods are also required. This will help gain insight into how end-users perceived the intervention, why they behaved the way they did and whether the intervention was effective in terms of behavior change (Dirican and Göktürk, 2011; Short *et al.*, 2018).

Currently, think-aloud studies and semi-structured interviews are being carried out in the context of Sense.info. Think-aloud procedures are particularly useful to understand cognitive processes and emotional reactions when participants are navigating the intervention and viewing intervention content (Short *et al.*, 2018; Newby *et al.*, 2019). Semi-structured interviews are used in addition to elaborate further on perceptions and statements made during the think-aloud procedure (Perski *et al.*, 2017a). For example, after having visited the chlamydia page whilst thinking aloud, participants are being interviewed and asked to elaborate on whether the provided information has changed their perceptions and motivations toward getting tested, and what changes would have to be made to reach greater impact in this respect. In other words, combining ABCDs and web analytics with end-user data leads to an even more detailed insight into potential impact of a web-based intervention.

In sum, this article shows how use of ABCDs in combination with use data analysis can lead to a more meaningful evaluation of web-based interventions. This is highly relevant given the current debate how engagement in web-based interventions should be measured. Although the ABCDs and web analytics tool Matomo were described in the context of a Dutch sexual health intervention, the underlying techniques can be used in evaluations of web-based interventions in general.

## SUPPLEMENTARY MATERIAL

Supplementary material is available at *Health Promotion International* online.

## Supplementary Material

daab190_Supplementary_Data
