# Supplementary material for: Evaluating use of web-based interventions: an example of a Dutch sexual health intervention
Source: Health Promot Int. 2021 Nov 22;38(4):daab190. doi: 10.1093/heapro/daab190 (PMC10439511; doi:10.1093/heapro/daab190)
Supplement: daab190_Supplementary_Data [file daab190_supplementary_data.zip › Supplementary file A.docx]

| Behavior Change Principles (BCPs) | Conditions for effectiveness | Applications | Sub-determinants (change objectives) | Determinants | Sub-behaviors (performance objectives | Target behavior |
| --- | --- | --- | --- | --- | --- | --- |
| Arguments | For central processing of arguments they need to be new to the message receiver. | Chlamydia page. Main text | Acknowledge that you should get tested for chlamydia if you have been at risk | Attitude | Testing for STIs | Having sex without STIs |
| Arguments | For central processing of arguments they need to be new to the message receiver. | Chlamydia page. Main text | Acknowledge you should seek treatment for chlamydia | Attitude | Seeking treatment for chlamydia | Having sex without STIs |
| Consciousness raising | Can use feedback and confrontation; however, raising awareness must be quickly followed by increase in problem-solving ability and (collective) self-efficacy. | Chlamydia page. Main text | Explain what chlamydia is | Knowledge | Testing for STIs | Having sex without STIs |
| Consciousness raising | Can use feedback and confrontation; however, raising awareness must be quickly followed by increase in problem-solving ability and (collective) self-efficacy. | Chlamydia page. Main text | Explain what chlamydia is | Knowledge | Seeking treatment for chlamydia | Having sex without STIs |
| Consciousness raising | Can use feedback and confrontation; however, raising awareness must be quickly followed by increase in problem-solving ability and (collective) self-efficacy. | Chlamydia page. Main text | List what the symptoms of chlamydia are | Knowledge | Testing for STIs | Having sex without STIs |
| Consciousness raising | Can use feedback and confrontation; however, raising awareness must be quickly followed by increase in problem-solving ability and (collective) self-efficacy. | Chlamydia page. Main text | List what the symptoms of chlamydia are | Knowledge | Seeking treatment for chlamydia | Having sex without STIs |
| Consciousness raising | Can use feedback and confrontation; however, raising awareness must be quickly followed by increase in problem-solving ability and (collective) self-efficacy. | Chlamydia page. Main text | State that you can have chlamydia without any symptoms | Risk perception | Testing for STIs | Having sex without STIs |
| Consciousness raising | Can use feedback and confrontation; however, raising awareness must be quickly followed by increase in problem-solving ability and (collective) self-efficacy. | Chlamydia page. Main text | State that you can have chlamydia without any symptoms | Risk perception | Seeking treatment for chlamydia | Having sex without STIs |
| Consciousness raising | Can use feedback and confrontation; however, raising awareness must be quickly followed by increase in problem-solving ability and (collective) self-efficacy. | Chlamydia page. Main text | State that treatment for chlamydia is easy | Knowledge | Testing for STIs | Having sex without STIs |
| Consciousness raising | Can use feedback and confrontation; however, raising awareness must be quickly followed by increase in problem-solving ability and (collective) self-efficacy. | Chlamydia page. Main text | State that treatment for chlamydia is easy | Knowledge | Seeking treatment for chlamydia | Having sex without STIs |
| Verbal persuasion | Credible source. | Chlamydia page. Main text | Express confidence that getting treated for chlamydia is easy | Self-efficacy | Testing for STIs | Having sex without STIs |
| Verbal persuasion | Credible source. | Chlamydia page. Main text | Express confidence that getting treated for chlamydia is easy | Self-efficacy | Seeking treatment for chlamydia | Having sex without STIs |
| Arguments | For central processing of arguments they need to be new to the message receiver. | Chlamydia page. Main text | Acknowledge that you should visit your General Practitioner or Sense consultation hour to get tested for STIs | Attitude | Testing for STIs | Having sex without STIs |
| Framing | Requires high self-efficacy expectations. Gain frames are more readily accepted and prevent defensive reactions. | Chlamydia page. Main text | List what can happen if you do not seek treatment | Risk perception | Seeking treatment for chlamydia | Having sex without STIs |
| Arguments | For central processing of arguments they need to be new to the message receiver. | Chlamydia page. Link to 'Partner notification' | Acknowledge that you should warn your partner(s) if you contracted an STI | Attitude | Warning your partner(s) that you contracted an STI | Having sex without STIs |
| Consciousness raising | Can use feedback and confrontation; however, raising awareness must be quickly followed by increase in problem-solving ability and (collective) self-efficacy. | Chlamydia page. Link to 'Partner notification' | Acknowledge that you should warn your partner(s) if you contracted an STI | Knowledge | Warning your partner(s) that you contracted an STI | Having sex without STIs |
| Arguments | For central processing of arguments they need to be new to the message receiver. | Chlamydia page. Link to 'STI-test' | Acknowledge you should get tested for STIs | Attitude | Testing for STIs | Having sex without STIs |
| Consciousness raising | Can use feedback and confrontation; however, raising awareness must be quickly followed by increase in problem-solving ability and (collective) self-efficacy. | Chlamydia page. Link to 'STI-test' | Explain why you should get tested for STIs | Knowledge | Testing for STIs | Having sex without STIs |
| Consciousness raising | Can use feedback and confrontation; however, raising awareness must be quickly followed by increase in problem-solving ability and (collective) self-efficacy. | Chlamydia page. Link to 'STI-test' | Explain where you can get tested for STIs | Knowledge | Testing for STIs | Having sex without STIs |
| Arguments | For central processing of arguments they need to be new to the message receiver. | Chlamydia page. Link to 'Safe sex' | Acknowledge that you should have safe sex | Attitude | Using condoms | Having sex without STIs |
| Consciousness raising | Can use feedback and confrontation; however, raising awareness must be quickly followed by increase in problem-solving ability and (collective) self-efficacy. | Chlamydia page. Link to 'Safe sex' | Acknowledge that you should have safe sex | Knowledge | Using condoms | Having sex without STIs |
| Consciousness raising | Can use feedback and confrontation; however, raising awareness must be quickly followed by increase in problem-solving ability and (collective) self-efficacy. | Chlamydia page. Link to 'Safe sex' | Explain how you can have safe sex | Skills | Using condoms | Having sex without STIs |
| Consciousness raising | Can use feedback and confrontation; however, raising awareness must be quickly followed by increase in problem-solving ability and (collective) self-efficacy. | Chlamydia page. Video about STIs | List what the symptoms of chlamydia are | Knowledge | Testing for STIs | Having sex without STIs |
| Consciousness raising | Can use feedback and confrontation; however, raising awareness must be quickly followed by increase in problem-solving ability and (collective) self-efficacy. | Chlamydia page. Video about STIs | List what the symptoms of chlamydia are | Knowledge | Seeking treatment for chlamydia | Having sex without STIs |
| Consciousness raising | Can use feedback and confrontation; however, raising awareness must be quickly followed by increase in problem-solving ability and (collective) self-efficacy. | Chlamydia page. Video about STIs | State that treatment for chlamydia is easy | Knowledge | Seeking treatment for chlamydia | Having sex without STIs |
| Consciousness raising | Can use feedback and confrontation; however, raising awareness must be quickly followed by increase in problem-solving ability and (collective) self-efficacy. | Chlamydia page. Video about STIs | State that you can have chlamydia without any symptoms | Risk perception | Testing for STIs | Having sex without STIs |
| Arguments | For central processing of arguments they need to be new to the message receiver. | Chlamydia page. Video about STIs | Acknowledge that you should visit your General Practitioner or Sense consultation hour to get tested for STIs | Attitude | Testing for STIs | Having sex without STIs |
| Framing | Requires high self-efficacy expectations. Gain frames are more readily accepted and prevent defensive reactions. | Chlamydia page. Video about STIs | List what can happen if you do not seek treatment | Risk perception | Seeking treatment for chlamydia | Having sex without STIs |
| Consciousness raising | Can use feedback and confrontation; however, raising awareness must be quickly followed by increase in problem-solving ability and (collective) self-efficacy. | Chlamydia page. Video about STIs | Explain what STIs are | Knowledge | Testing for STIs | Having sex without STIs |
| Consciousness raising | Can use feedback and confrontation; however, raising awareness must be quickly followed by increase in problem-solving ability and (collective) self-efficacy. | Chlamydia page. Link to 'What are STIs?' | Explain what STIs are | Knowledge | Testing for STIs | Having sex without STIs |
| Consciousness raising | Can use feedback and confrontation; however, raising awareness must be quickly followed by increase in problem-solving ability and (collective) self-efficacy. | Chlamydia page. Link to 'Genital warts' | Explain what genital warts are | Knowledge | Testing for STIs | Having sex without STIs |
| Consciousness raising | Can use feedback and confrontation; however, raising awareness must be quickly followed by increase in problem-solving ability and (collective) self-efficacy. | Chlamydia page. Link to 'Herpes Genitalis' | Explain what herpes genitalis is | Knowledge | Testing for STIs | Having sex without STIs |
| Consciousness raising | Can use feedback and confrontation; however, raising awareness must be quickly followed by increase in problem-solving ability and (collective) self-efficacy. | Chlamydia page. Link to 'Gonorrhea' | Explain what gonorrhea is | Knowledge | Testing for STIs | Having sex without STIs |
| Consciousness raising | Can use feedback and confrontation; however, raising awareness must be quickly followed by increase in problem-solving ability and (collective) self-efficacy. | Chlamydia page. Link to 'Hepatitis B' | Explain what hepatitis B is | Knowledge | Testing for STIs | Having sex without STIs |
| Consciousness raising | Can use feedback and confrontation; however, raising awareness must be quickly followed by increase in problem-solving ability and (collective) self-efficacy. | Chlamydia page. Link to 'Syphilis' | Explain what syphilis is | Knowledge | Testing for STIs | Having sex without STIs |
| Consciousness raising | Can use feedback and confrontation; however, raising awareness must be quickly followed by increase in problem-solving ability and (collective) self-efficacy. | Chlamydia page. Link to 'Bacterial vaginosis' | Explain what bacterial vaginosis is | Knowledge | Testing for STIs | Having sex without STIs |
| Consciousness raising | Can use feedback and confrontation; however, raising awareness must be quickly followed by increase in problem-solving ability and (collective) self-efficacy. | Chlamydia page. Link to 'Candida-infection' | Explain what candida infection is | Knowledge | Testing for STIs | Having sex without STIs |
| Consciousness raising | Can use feedback and confrontation; however, raising awareness must be quickly followed by increase in problem-solving ability and (collective) self-efficacy. | Chlamydia page. Link to 'Scabies' | Explain what scabies is | Knowledge | Testing for STIs | Having sex without STIs |
| Consciousness raising | Can use feedback and confrontation; however, raising awareness must be quickly followed by increase in problem-solving ability and (collective) self-efficacy. | Chlamydia page. Link to 'Pubic lice' | Explain what pubic lice is | Knowledge | Testing for STIs | Having sex without STIs |
| Consciousness raising | Can use feedback and confrontation; however, raising awareness must be quickly followed by increase in problem-solving ability and (collective) self-efficacy. | Chlamydia page. Link to 'Trichomonas' | Explain what trichomonas is | Knowledge | Testing for STIs | Having sex without STIs |
| Consciousness raising | Can use feedback and confrontation; however, raising awareness must be quickly followed by increase in problem-solving ability and (collective) self-efficacy. | Chlamydia page. Link to 'Hiv' | Explain what hiv is | Knowledge | Testing for STIs | Having sex without STIs |
| Modeling | Attention, remembrance, self- efficacy and skills, reinforcement of model; identification with model, coping model instead of mastery model. | Chlamydia page. Video 'Marjan tells her boyfriend she contracted chlamydia' | Acknowledge that you should warn your partner(s) if you contracted an STI | Attitude | Warning your partner(s) that you contracted an STI | Having sex without STIs |
| Modeling | Attention, remembrance, self- efficacy and skills, reinforcement of model; identification with model, coping model instead of mastery model. | Chlamydia page. Video 'Marjan tells her boyfriend she contracted chlamydia' | Acknowledge that you should warn your partner(s) if you contracted an STI | Attitude | Seeking treatment for chlamydia | Having sex without STIs |
| Modeling | Attention, remembrance, self- efficacy and skills, reinforcement of model; identification with model, coping model instead of mastery model. | Chlamydia page. Video 'Marjan tells her boyfriend she contracted chlamydia' | Explain why you should warn your partner(s) if you contracted an STI | Knowledge | Warning your partner(s) that you contracted an STI | Having sex without STIs |
| Modeling | Attention, remembrance, self- efficacy and skills, reinforcement of model; identification with model, coping model instead of mastery model. | Chlamydia page. Video 'Marjan tells her boyfriend she contracted chlamydia' | Explain how you can warn your partner(s) if you contracted an STI | Skills | Warning your partner(s) that you contracted an STI | Having sex without STIs |
| Modeling | Attention, remembrance, self- efficacy and skills, reinforcement of model; identification with model, coping model instead of mastery model. | Chlamydia page. Video 'Marjan tells her boyfriend she contracted chlamydia' | Acknowledge you should take a warning from your partner seriously | Attitude | Testing for STIs | Having sex without STIs |
| Consciousness raising | Can use feedback and confrontation; however, raising awareness must be quickly followed by increase in problem-solving ability and (collective) self-efficacy. | Chlamydia page. Video by Soa-Aids Nederland about chlamydia | List what the symptoms of chlamydia are | Knowledge | Testing for STIs | Having sex without STIs |
| Consciousness raising | Can use feedback and confrontation; however, raising awareness must be quickly followed by increase in problem-solving ability and (collective) self-efficacy. | Chlamydia page. Video by Soa-Aids Nederland about chlamydia | List what the symptoms of chlamydia are | Knowledge | Seeking treatment for chlamydia | Having sex without STIs |
| Consciousness raising | Can use feedback and confrontation; however, raising awareness must be quickly followed by increase in problem-solving ability and (collective) self-efficacy. | Chlamydia page. Video by Soa-Aids Nederland about chlamydia | Explain how chlamydia is being treated | Knowledge | Testing for STIs | Having sex without STIs |
| Consciousness raising | Can use feedback and confrontation; however, raising awareness must be quickly followed by increase in problem-solving ability and (collective) self-efficacy. | Chlamydia page. Video by Soa-Aids Nederland about chlamydia | Explain how chlamydia is being treated | Knowledge | Seeking treatment for chlamydia | Having sex without STIs |
| Consciousness raising | Can use feedback and confrontation; however, raising awareness must be quickly followed by increase in problem-solving ability and (collective) self-efficacy. | Chlamydia page. Video by Soa-Aids Nederland about chlamydia | Explain how to get tested for chlamydia | Knowledge | Testing for STIs | Having sex without STIs |
| Consciousness raising | Can use feedback and confrontation; however, raising awareness must be quickly followed by increase in problem-solving ability and (collective) self-efficacy. | Chlamydia page. Video by Soa-Aids Nederland about chlamydia | Explain how to get tested for chlamydia | Knowledge | Seeking treatment for chlamydia | Having sex without STIs |
| Consciousness raising | Can use feedback and confrontation; however, raising awareness must be quickly followed by increase in problem-solving ability and (collective) self-efficacy. | Chlamydia page. Video by Soa-Aids Nederland about chlamydia | Explain how chlamydia can be prevented | Knowledge | Using condoms | Having sex without STIs |
| Consciousness raising | Can use feedback and confrontation; however, raising awareness must be quickly followed by increase in problem-solving ability and (collective) self-efficacy. | Chlamydia page. Video by Soa-Aids Nederland about chlamydia | Explain what chlamydia is | Knowledge | Testing for STIs | Having sex without STIs |
| Consciousness raising | Can use feedback and confrontation; however, raising awareness must be quickly followed by increase in problem-solving ability and (collective) self-efficacy. | Chlamydia page. Video by Soa-Aids Nederland about chlamydia | Explain what chlamydia is | Knowledge | Seeking treatment for chlamydia | Having sex without STIs |
| Consciousness raising | Can use feedback and confrontation; however, raising awareness must be quickly followed by increase in problem-solving ability and (collective) self-efficacy. | Chlamydia page. Video by Soa-Aids Nederland about chlamydia | State that you can have chlamydia without any symptoms | Knowledge | Testing for STIs | Having sex without STIs |
| Consciousness raising | Can use feedback and confrontation; however, raising awareness must be quickly followed by increase in problem-solving ability and (collective) self-efficacy. | Chlamydia page. Video by Soa-Aids Nederland about chlamydia | State that you can have chlamydia without any symptoms | Knowledge | Seeking treatment for chlamydia | Having sex without STIs |
| Consciousness raising | Can use feedback and confrontation; however, raising awareness must be quickly followed by increase in problem-solving ability and (collective) self-efficacy. | Chlamydia page. Video by Soa-Aids Nederland about chlamydia | State that treatment for chlamydia is easy | Knowledge | Testing for STIs | Having sex without STIs |
| Consciousness raising | Can use feedback and confrontation; however, raising awareness must be quickly followed by increase in problem-solving ability and (collective) self-efficacy. | Chlamydia page. Video by Soa-Aids Nederland about chlamydia | State that treatment for chlamydia is easy | Knowledge | Seeking treatment for chlamydia | Having sex without STIs |
| Consciousness raising | Can use feedback and confrontation; however, raising awareness must be quickly followed by increase in problem-solving ability and (collective) self-efficacy. | Chlamydia page. Video by Soa-Aids Nederland about chlamydia | Acknowledge that you should visit your General Practitioner or Sense consultation hour to get tested for STIs | Knowledge | Testing for STIs | Having sex without STIs |
| Framing | Requires high self-efficacy expectations. Gain frames are more readily accepted and prevent defensive reactions. | Chlamydia page. Video by Soa-Aids Nederland about chlamydia | List what can happen if you do not seek treatment | Knowledge | Testing for STIs | Having sex without STIs |
| Framing | Requires high self-efficacy expectations. Gain frames are more readily accepted and prevent defensive reactions. | Chlamydia page. Video by Soa-Aids Nederland about chlamydia | List what can happen if you do not seek treatment | Knowledge | Seeking treatment for chlamydia | Having sex without STIs |
| Consciousness raising | Can use feedback and confrontation; however, raising awareness must be quickly followed by increase in problem-solving ability and (collective) self-efficacy. | Chlamydia page. Video by Soa-Aids Nederland about chlamydia | Acknowledge that you should warn your partner(s) if you contracted an STI | Knowledge | Warning your partner(s) that you contracted an STI | Having sex without STIs |
