# Supplementary figures and images for: Evaluating use of web-based interventions: an example of a Dutch sexual health intervention
Source: Health Promot Int. 2021 Nov 22;38(4):daab190. doi: 10.1093/heapro/daab190 (PMC10439511; doi:10.1093/heapro/daab190)

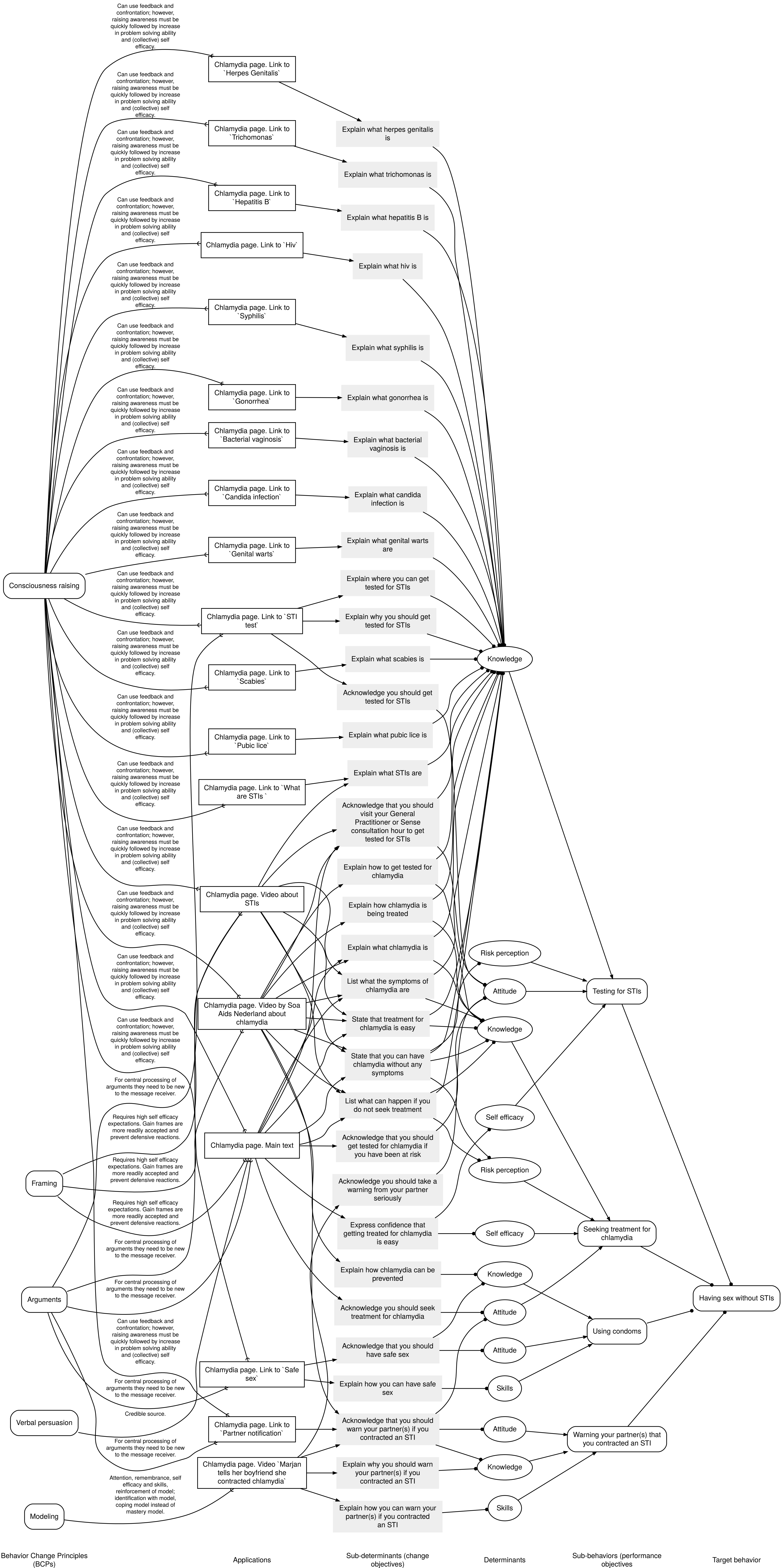

Supplement: daab190_Supplementary_Data [file daab190_supplementary_data.zip › Supplementary file B.pdf]
